# A Comparison of Portable Ultrasound and Fully-Equipped Clinical Ultrasound Unit in the Thyroid Size Measurement of the Indo-Pacific Bottlenose Dolphin

**DOI:** 10.1371/journal.pone.0030218

**Published:** 2012-01-17

**Authors:** Brian C. W. Kot, Michael T. C. Ying, Fiona M. Brook

**Affiliations:** Department of Health Technology and Informatics, The Hong Kong Polytechnic University, Hung Hom, Hong Kong SAR, China; University of Georgia, United States of America

## Abstract

Measurement of thyroid size and volume is a useful clinical parameter in both human and veterinary medicine, particularly for diagnosing thyroid diseases and guiding corrective therapy. Procuring a fully-equipped clinical ultrasound unit (FCUS) may be difficult in most veterinary settings. The present study evaluated the inter-equipment variability in dolphin thyroid ultrasound measurements between a portable ultrasound unit (PUS) and a FCUS; for both units, repeatability was also assessed. Thyroid ultrasound examinations were performed on 15 apparently healthy bottlenose dolphins with both PUS and FCUS under identical scanning conditions. There was a high level of agreement between the two ultrasound units in dolphin thyroid measurements (ICC = 0.859–0.976). A high intra-operator repeatability in thyroid measurements was found (PUS: ICC = 0.854–0.984, FCUS: ICC = 0.709–0.954). As a conclusion, no substantial inter-equipment variability was found between PUS and FCUS in dolphin thyroid size measurements under identical scanning conditions, supporting further application of PUS for quantitative analyses of dolphin thyroid gland in both research and clinical practices at aquarium settings.

## Introduction

Ultrasound is a non-invasive, real-time imaging tool that provides high resolution images for soft tissue characterization, and allows repeatable measurements. 2-D ultrasound has a prominent role in evaluating the morphology of the thyroid gland in humans [1]–[3] and companion animals [4]–[7]. The mammalian thyroid gland is critical in regulating metabolic functions including cardiac rate and output, lipid catabolism, skeletal growth, and production of oxygen and heat. Environmental contaminants and local environmental influences have been implicated in thyroid hormone imbalances [8] and development of morphological and histological abnormalities [9]–[11] leading to calf mortality [12]. To the best of our knowledge, the formal literature is devoid of any reference to the diagnosis of thyroid abnormalities in living dolphins. In order to accurately diagnose and assess thyroid abnormalities in live animals, reliable methods of assessing the thyroid morphology must be developed so that corrective therapy can be undertaken.

In human medicine, the thyroid volume is a useful clinical measure, particularly in the diagnosis of thyroid diseases and accurate determination of the iodine-131 dosage used in radioiodine therapy for hyperthyroidism. Volume measurement of each lobe is usually estimated using the ellipsoid equation [13] i.e. volume = π/6× craniocaudal × mediolateral × dorsoventral dimensions and its derivatives using the cross-sectional area [14]. Recently, efforts have been made to establish a standardized scanning protocol in evaluating the morphology of the thyroid gland in a group of Indo-Pacific bottlenose dolphins using a fully-equipped clinical ultrasound unit (FCUS) with 3-D ultrasound capabilities [15]. Using these equations [13], [14], 4 ultrasound thyroid volume measurement methods (Methods A–D) were developed, in which 13 linear and 5 cross-sectional measurements were undertaken in the dolphin thyroid study. Since serial ultrasound measurements of the dimensions of thyroid gland have been proven to be useful in identifying thyroid diseases and monitoring treatment response [1], [16], [17], assessment of the aforementioned dimensions of the dolphin thyroid gland is essential, in addition to the thyroid volume itself.

Access to a FCUS, as well as 3-D ultrasound equipment, may be limited at zoological and aquarium settings. Procuring a FCUS is not always feasible in most veterinary settings due to its high start-up and maintenance cost. In addition, its bulkiness makes it unfavourable in various captive animal settings. A portable ultrasound unit (PUS) equipped with basic ultrasound functions for veterinary medicine has a comparatively lower cost that is affordable for most zoological and aquarium settings. Ultrasound studies conducted in various veterinary clinical settings, as well as wildlife research projects, have been mostly performed with different PUSs [18], [19]. However, the miniaturization of the PUS is believed to create compromises in function, and there are concerns regarding the image quality in these smaller and less expensive units. In view of the presently extensive applications of PUS in veterinary imaging, from being a diagnostic tool for routine clinical check-up of a range of species, to conducting disease screening, conservation projects, commercial services, herd management and clinical research, it is important to evaluate the inter-equipment variability between the PUS and FCUS in terms of direct linear measurements as well as cross-sectional areas of specific planes, which are essential parameters for volume measurement of an interested organ. In addition, the intra-operator variability (repeatability) of the individual PUS and FCUS should be further examined under the same scanning conditions to ensure accurate assessments of the thyroid size in follow-up examinations throughout the course of treatment.

The aims of the present study were to evaluate the inter-equipment variability in dolphin thyroid ultrasound linear and cross-sectional area measurements between a PUS (Aloka SSD 900) and a FCUS (Philips HD 11) under identical scanning conditions, and to assess the repeatability of these measurements using both ultrasound units.

## Methods

### Subjects and Study Design

Fifteen *Tursiops aduncus* at Ocean Park, Hong Kong (5 males and 10 females) were included in the study. The mean age of the subjects was 15.1 years (range, 2–35 years). Diets consisted of different proportions of capelin, sardine, herring and squid, along with vitamin and mineral supplements. The subjects were apparently healthy with no recent history of illnesses, and were not receiving medication that could alter thyroid gland physiology during the time of the study. Serum concentrations of thyroxine (free [fT4] and total [tT4]), triiodothyronine (free [fT3], total [tT3]) were also determined on each individual subject and the values were all within normal ranges [20]. All dolphins involved in the study were trained to cooperate for neck ultrasound examination. Ultrasound images from each dolphin were taken on its thyroid using a PUS Aloka SSD 900 ultrasound unit in conjunction with a 5 MHz curvilinear transducer (Aloka Company Ltd., Tokyo, Japan) and a FCUS Philips HD 11 ultrasound unit in conjunction with a 5−2 MHz broadband curved array transducer (Philips Medical System, Bothell, Washington, 98021, USA).

### Technical Differences between the PUS and the FCUS

The Aloka SSD 900 ultrasound unit is a miniaturized portable general imaging ultrasound unit that provides 256 shades of gray resolution and dynamic focus. This PUS is more portable than the FCUS because of its comparatively small size and low weight (13.6 kg). Similar to the FCUS, the PUS also offers a full range of measurement functions for clinical ultrasound examinations and incorporates super high density transducers to enhance imaging resolution.

Technical details of the PUS and the FCUS that may influence the thyroid linear and cross-sectional area measurements are listed (Table 1).

**Table 1:** Technical details of the portable ultrasound unit (PUS) and the fully-equipped clinical ultrasound unit (FCUS).

	Ultrasound Machine	
Technical details	PUS	FCUS
Transducer frequency (MHz)	5	5–2
Frame rate (frames per second)	max 237	max 785
Gain setting	operator defined	operator defined
Grey scale	operator defined	operator defined
Persistence setting	4 settings	7 settings
Number of depth settings	11	30
Number of focus settings	4 user-selectable focal zones	4 user-selectable focal zones
Image resolution (axial resolution)	At 5 cm depth: 1 mm; At 11 cm depth: 1 mm	At 5 cm depth: 1 mm; At 11 cm depth: 1 mm
	At 5 MHz	At 4.25 MHz (centre frequency)
Image resolution (lateral resolution)	At 5 cm depth: 2 mm; At 11 cm depth: 4 mm	At 5 cm depth: 2 mm; At 11 cm depth: 4 mm
	At 5 MHz	At 4.25 MHz (centre frequency)

### Thyroid Ultrasound Imaging and Measurement

Ultrasound measurements using both units were performed by the same operator (BK) and the operator was blinded to the linear and cross-sectional area measurements obtained from both units. There was a time interval of at least 30 minutes between measurements of the 2 sets of images from the same dolphin thyroid gland. Therefore, recall bias of the results for the same dolphin thyroid gland was avoided. The operator had more than 3 years of experience in performing dolphin thyroid ultrasound examinations. Standardized scanning protocol for dolphin thyroid gland was used in the present study [15]. Four 2-D ultrasound thyroid volume measurement methods (Methods A–D) were developed using the ellipsoid equation [13] i.e. volume = π/6× craniocaudal × mediolateral × dorsoventral dimensions; and its derivatives using the cross-sectional area is shown (Table 2) [14]. Detailed linear and cross-sectional area measurements were undertaken as described below.

**Table 2:** Equations of each method for calculating the thyroid volume.

Method	Equation for calculation of thyroid volume
A (2-D US^f^)	π/6× TS_MAX^a^ × mean of craniocaudal dimension in 3 planes (LS_L^b^, LS_MID^c^ and LS_R^d^) × mean of dorsoventral dimension in 3 planes (LS_L^b^, LS_MID^c^ and LS_R^d^)
B (2-D US^f^)	2/3× TS_MAX^a^ × mean of cross-sectional area of 3 planes (LS_L^b^, LS_MID^c^ and LS_R^d^)
C (2-D US^f^)	π/6× craniocaudal × mediolateral × dorsoventral
D (2-D US^f^)	2/3× craniocaudal × maximum cross-sectional area^e^
E (3-D US^g^)	Calculated by in-built software (QLAB, Philips)

a The maximum transverse dimension of the thyroid gland.

b The maximum longitudinal scan plane of the left thyroid lobe.

c The longitudinal scan plane of the midline of the thyroid gland.

d The maximum longitudinal scan plane of the right thyroid lobe.

e π/4× mediolateral × dorsoventral.

f Two-dimensional ultrasound.

g Three-dimensional ultrasound.

### Methods A and B

**Figure 1:**
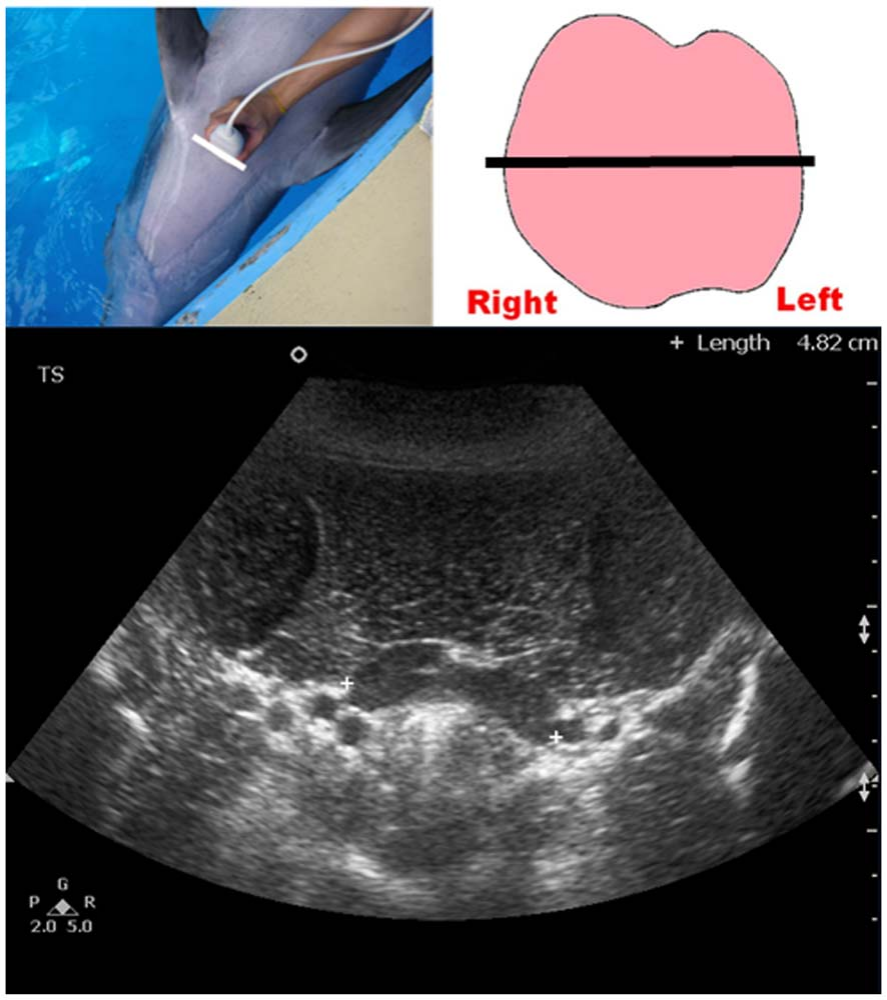
Ultrasound measurement of the maximum transverse dimension of the dolphin thyroid gland (TS_MAX). Top left picture shows the position of the transducer at the neck region. Top right picture shows the schematic diagram of the thyroid gland in a dorsal orientation with the straight line representing the position of the transducer. Bottom image shows a transverse grey scale sonogram of the thyroid gland of a bottlenose dolphin. Note the maximum transverse dimension of the thyroid gland is measured (calipers +).

**Figure 2:**
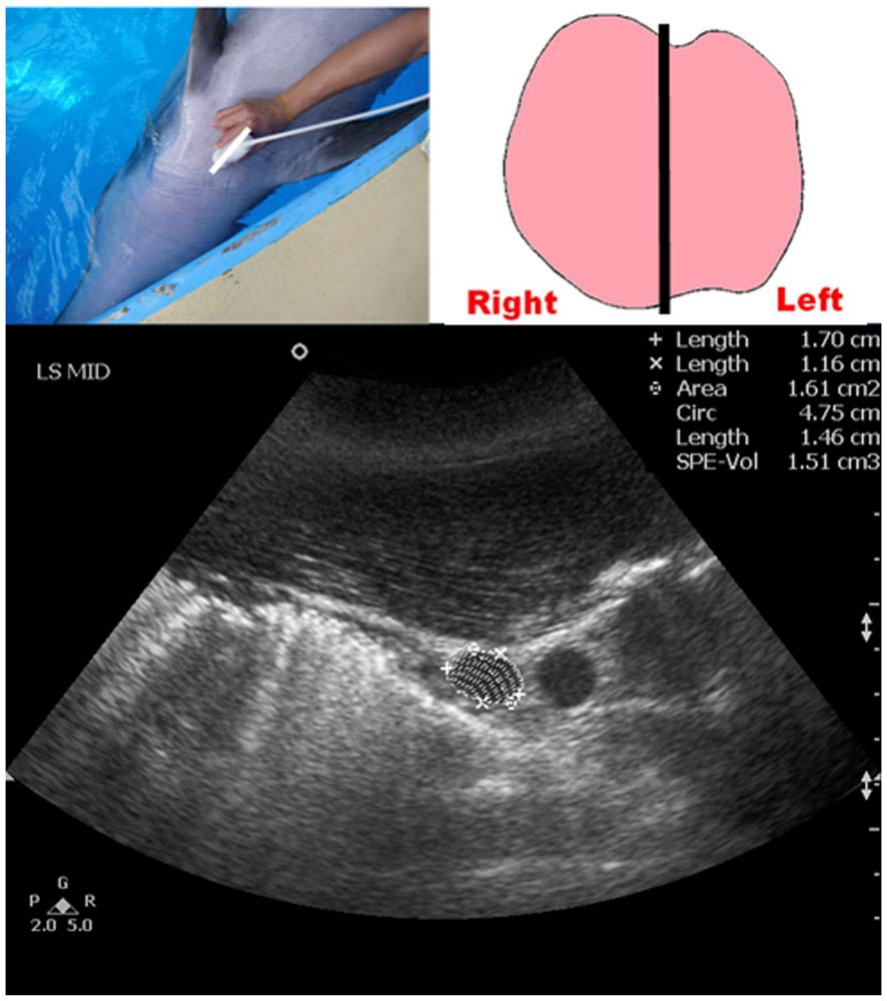
Ultrasound measurement of the longitudinal dimension of the dolphin thyroid gland at the midline (LS_MID). Top left picture shows the position of the transducer at the neck region. Top right picture shows the schematic diagram of the thyroid gland in a dorsal orientation with the straight line representing the position of the transducer. Bottom image shows a longitudinal grey scale sonogram of the thyroid gland of a bottlenose dolphin. Note the dorsoventral dimension (calipers x), the craniocaudal dimension (calipers +) and the cross-sectional area (dotted line) are measured respectively.

**Figure 3:**
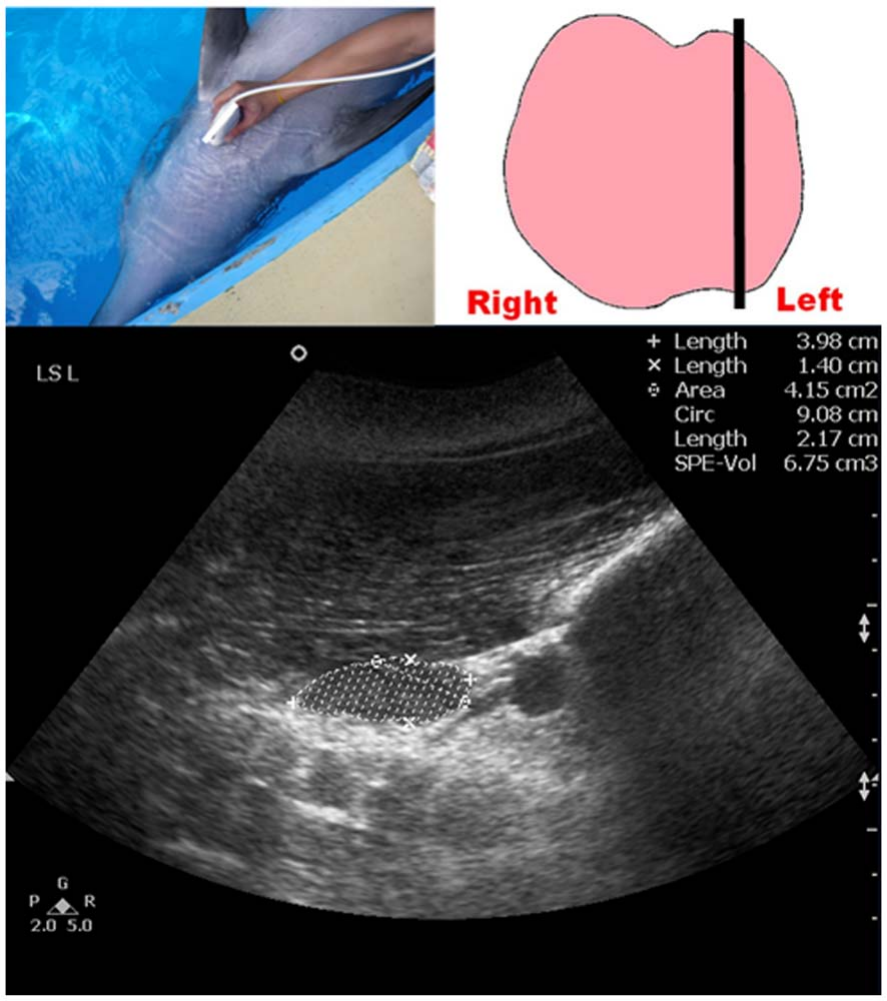
Ultrasound measurement of the maximum longitudinal dimension of the left thyroid lobe of a dolphin (LS_L). Top left picture shows the position of the transducer at the neck region. Top right picture shows the schematic diagram of the thyroid gland in a dorsal orientation with the straight line representing the position of the transducer. Bottom image shows a longitudinal grey scale sonogram of the left thyroid lobe of a bottlenose dolphin. Note the maximum longitudinal dimension of the left thyroid lobe is demonstrated, and the dorsoventral dimension (calipers x), the craniocaudal dimension (calipers +) and the cross-sectional area (dotted line) are measured respectively. The same ultrasound measurement of the maximum longitudinal dimension was repeated on the right thyroid lobe.

**Figure 4:**
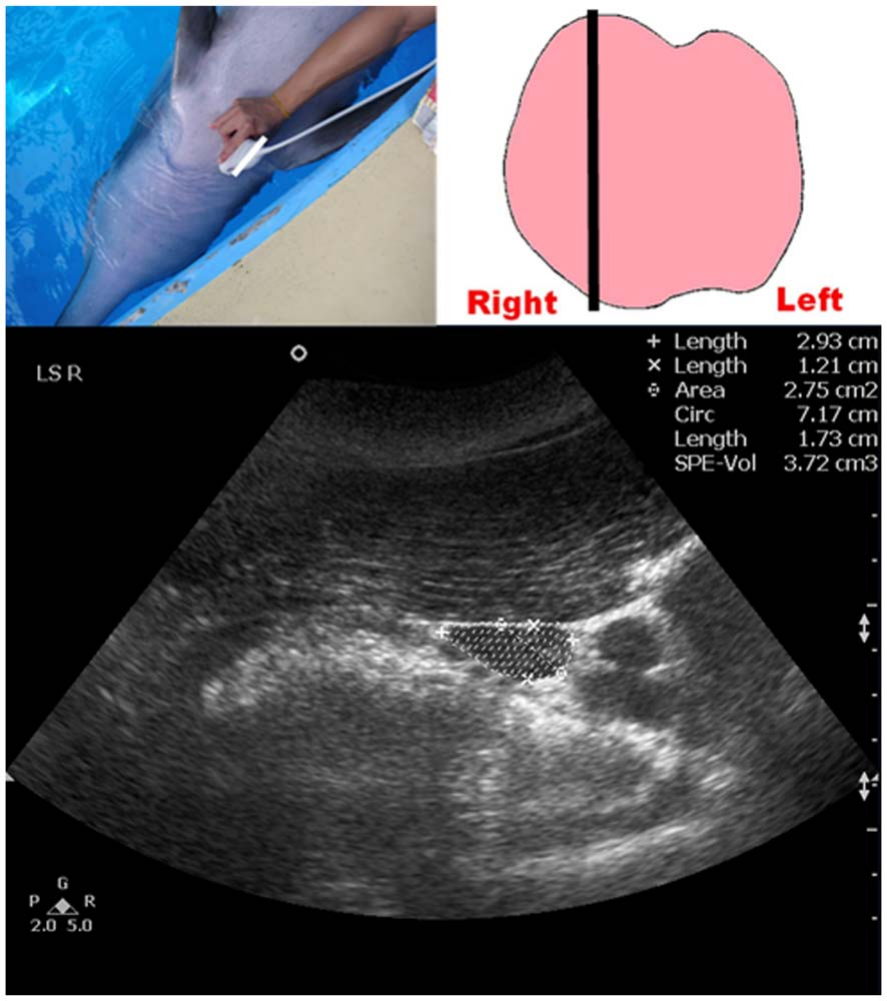
Ultrasound measurement of the maximum longitudinal dimension of the right thyroid lobe of a dolphin (LS_R). Top left picture shows the position of the transducer at the neck region. Top right picture shows the schematic diagram of the thyroid gland in a dorsal orientation with the straight line representing the position of the transducer. Bottom image shows a longitudinal grey scale sonogram of the right thyroid lobe of a bottlenose dolphin. Note the maximum longitudinal dimension of the right thyroid lobe is demonstrated, and the dorsoventral dimension (calipers x), the craniocaudal dimension (calipers +) and the cross-sectional area (dotted line) are measured respectively.

Once the location of the thyroid gland was identified, the transducer was then moved cranially and caudally until the scan plane showing the maximum transverse dimension of the thyroid gland (TS_MAX) was obtained and the TS_MAX was then measured (Figure 1). The transducer was then rotated 90°, to show the longitudinal scan planes of the thyroid gland. A full survey of the thyroid gland was performed in the longitudinal scan with the transducer moved from the left lobe to the right lobe. Images of the three longitudinal scan planes were recorded (Figures 2, 3, 4): 1. scan plane showing the midline of the thyroid gland (LS_MID); 2. scan plane showing the maximum longitudinal dimension of the left lobe (LS_L); 3. scan plane showing the maximum longitudinal dimension of the right lobe (LS_R). In each longitudinal scan plane, the dorsoventral dimension, the craniocaudal dimension, and the cross-sectional area of the thyroid lobe were measured.

### Methods C and D

**Figure 5:**
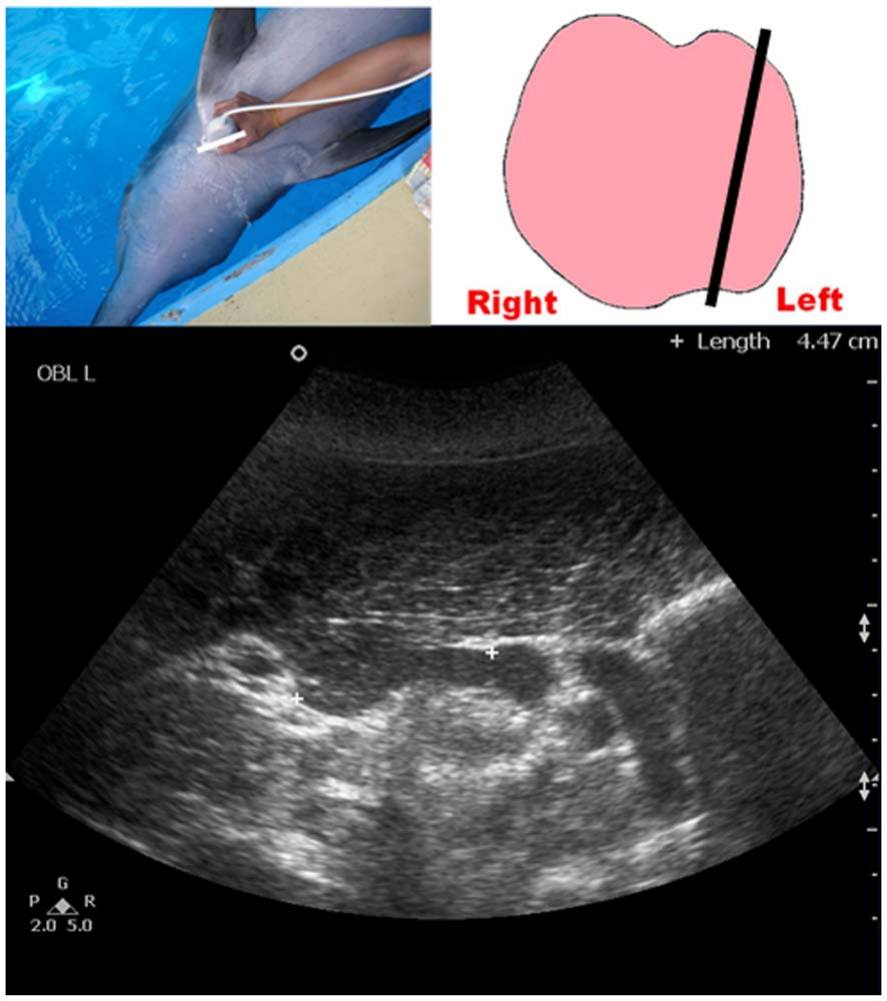
Ultrasound measurement of the long axis of the left thyroid lobe of a dolphin. Top left picture shows the position of the transducer at the neck region. Top right picture shows the schematic diagram of the thyroid gland in a dorsal orientation with the straight line representing the position of the transducer. Bottom image shows an oblique grey scale sonogram of the left thyroid lobe of a bottlenose dolphin. Note the long axis of the left thyroid lobe is measured (calipers +).

**Figure 6:**
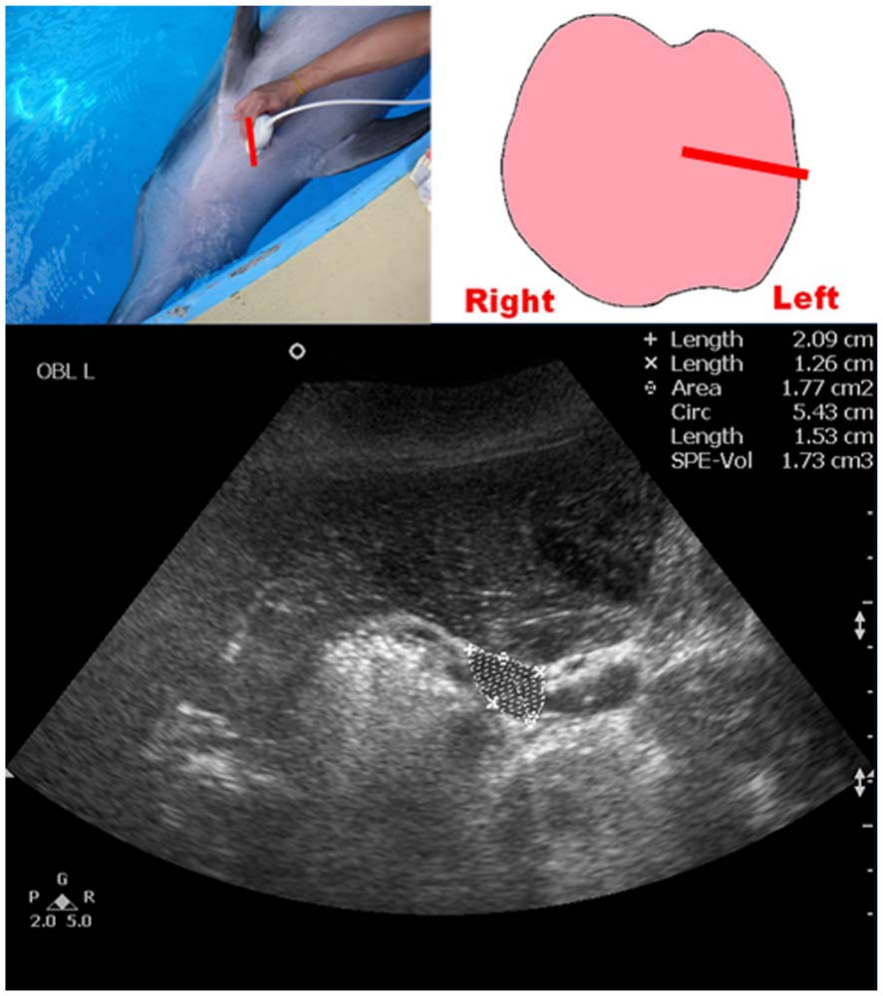
Ultrasound measurement of the maximum cross-sectional area of the left thyroid lobe of a dolphin. Top left picture shows the position of the transducer at the neck region. Top right picture shows the schematic diagram of the thyroid gland in a dorsal orientation with the straight line representing the position of the transducer. Bottom image shows an oblique grey scale sonogram of the left thyroid lobe of a bottlenose dolphin. Note the maximum cross-sectional area of the left thyroid lobe is demonstrated, and the dorsoventral dimension (calipers x), the mediolateral dimension (calipers +) and the cross-sectional area (dotted line) are measured respectively.

The transducer was initially placed obliquely on one side of the thyroid gland and then the transducer was slightly rotated clockwise and anticlockwise until the image showing the longest axis of the thyroid lobe was identified and recorded. The long axis of the thyroid lobe was then measured (Figure 5). The transducer was then rotated 90° to show the cross-sectional image of the thyroid lobe. A full survey of the cross-sectional image of the thyroid lobe was performed by scanning from the upper to lower poles of the thyroid gland. The scan plane showing the maximum cross-sectional area of the thyroid lobe was recorded, and the dorsoventral dimension, the mediolateral diameter and the cross-sectional area of the thyroid lobe were measured (Figure 6). The same scanning protocol was repeated for the contralateral thyroid lobe.

During the thyroid scanning with each ultrasound unit, time-gain-compensation and depth settings were adjusted to optimize image quality. For both ultrasound units, all measurements were performed using the electronic calipers. For the Aloka SSD 900 ultrasound unit, all images were recorded onto thermal printing paper, scanned and stored into digital format, while the images obtained by the Philips HD 11 were captured and stored digitally.

### Statistical Analysis

To analyze the inter-equipment variability of both ultrasound units, different thyroid ultrasound linear and cross-sectional area measurements were assessed by the intraclass correlation coefficient (ICC) and 95% confidence intervals (C.I.). In order to evaluate the intra-operator variability (repeatability) of the different thyroid ultrasound linear and cross-sectional area measurements, intraclass correlation coefficient (ICC) and 95% C.I. were also used to assess the level of agreement of the measurements in a single operator (BK). An ICC>0.7 is commonly used to indicate sufficient general reliability [21], [22]. All statistical analyses were carried out using SPSS (SPSS for windows 16.0, SPSS Inc., Chicago, Illinois).

This study was licensed under the Animals Control of Experiments Ordinance, Cap 340, issued by the Department of Health of Hong Kong Special Administrative Region. All procedures were reviewed and approved by the Animal Subjects Ethics Sub-committee of the Hong Kong Polytechnic University and the Scientific Advisory Committee of Ocean Park Hong Kong.

## Results

The inter-equipment variability of the different thyroid ultrasound linear and cross-sectional area measurements is shown (Table 3). Overall, the ICC was 0.964 with 95% C.I. range of 0.889–0.988. Results demonstrated that the ICC values of all measurements were above 0.85, indicating correlations of over 85% between both ultrasound units. The cross-sectional area measurements yielded a higher inter-equipment reproducibility than the linear measurements. Overall, both ultrasound units yielded a high level of agreement in different thyroid ultrasound linear and cross-sectional area measurements.

**Table 3:** Inter-equipment variability of the ultrasound thyroid linear and cross-sectional area measurements.

Measurement	ICC^k^ (2,1)	95% C.I.^l^ of ICC^k^ (Lower - Upper)
Max TS^a^	0.969	0.896–0.990
L LS^b^ (H^h^)	0.907	0.752–0.967
L LS^b^ (W^i^)	0.915	0.766–0.971
L LS^b^ (CSA^j^)	0.934	0.821–0.977
Mid LS^c^ (H^h^)	0.939	0.829–0.979
Mid LS^c^ (W^i^)	0.938	0.801–0.980
Mid LS^c^ (CSA^j^)	0.976	0.894–0.993
R LS^d^ (H^h^)	0.958	0.818–0.987
R LS^d^ (W^i^)	0.933	0.813–0.977
R LS^d^ (CSA^j^)	0.949	0.648–0.987
L Obl^e^ (L^g^)	0.943	0.819–0.981
L Obl^e^ (H^h^)	0.936	0.824–0.978
L Obl^e^ (W^i^)	0.877	0.677–0.957
L Obl^e^ (CSA^j^)	0.949	0.859–0.982
R Obl^f^ (L^g^)	0.924	0.796–0.974
R Obl^f^ (H^h^)	0.859	0.638–0.950
R Obl^f^ (W^i^)	0.925	0.758–0.976
R Obl^f^ (CSA^j^)	0.959	0.884–0.986

a The maximum transverse dimension of the thyroid gland.

b The maximum longitudinal scan plane of the left thyroid lobe.

c The longitudinal scan plane of the left thyroid lobe.

d The maximum longitudinal scan plane of the right thyroid lobe.

e The oblique scan plane of the left thyroid lobe.

f The oblique scan plane of the right thyroid lobe.

g Length; craniocaudal dimension.

h Height; dorsoventral dimension.

i Width; mediolateral dimension.

j Cross-sectional area.

k Intraclass Correlation Coefficient.

l Confidence Interval.

The intra-operator variability (repeatability) of using the 2 ultrasound units in thyroid ultrasound linear and cross-sectional area measurements is shown (Table 4). Overall, the ICC was 0.974 with 95% C.I. range of 0.925–0.991 for the PUS and 0.962 with 95% C.I. range of 0.891–0.987 for the FCUS. The cross-sectional area measurements yielded a higher intra-operator repeatability than the linear measurements. Results demonstrated that both ultrasound units yielded a high intra-operator repeatability for all thyroid ultrasound linear and cross-sectional area measurements. Compared to the FCUS, the PUS showed a higher repeatability.

**Table 4:** Intra-operator (repeatability) variability of the ultrasound thyroid linear and cross-sectional area measurements.

Measurement	PUS^m^	FCUS^n^		
	ICC^k^(3,1)	95% C.I.^l^ of ICC^k^(Lower - Upper)	ICC^k^ (3,1)	95% C.I.^l^ of ICC^k^(Lower - Upper)
Max TS^a^	0.974	0.924–0.991	0.954	0.870–0.984
L LS^b^ (H^h^)	0.949	0.854–0.982	0.722	0.351–0.897
L LS^b^ (W^i^)	0.890	0.705–0.962	0.863	0.640–0.952
L LS^b^ (CSA^j^)	0.927	0.797–0.975	0.904	0.738–0.967
Mid LS^c^ (H^h^)	0.965	0.900–0.988	0.856	0.624–0.949
Mid LS^c^ (W^i^)	0.914	0.765–0.970	0.835	0.577–0.941
Mid LS^c^ (CSA^j^)	0.981	0.945–0.994	0.884	0.691–0.960
R LS^d^ (H^h^)	0.973	0.921–0.991	0.887	0.697–0.961
R LS^d^ (W^i^)	0.854	0.619–0.948	0.851	0.613–0.947
R LS^d^ (CSA^j^)	0.974	0.925–0.991	0.951	0.861–0.983
L Obl^e^ (L^g^)	0.984	0.952–0.994	0.867	0.650–0.953
L Obl^e^ (H^h^)	0.934	0.815–0.977	0.898	0.724–0.964
L Obl^e^ (W^i^)	0.928	0.800–0.975	0.878	0.676–0.957
L Obl^e^ (CSA^j^)	0.956	0.873–0.985	0.928	0.799–0.875
R Obl^f^ (L^g^)	0.950	0.857–0.983	0.939	0.829–0.979
R Obl^f^ (H^h^)	0.930	0.806–0.976	0.709	0.327–0.892
R Obl^f^ (W^i^)	0.896	0.720–0.964	0.802	0.508–0.929
R Obl^f^ (CSA^j^)	0.975	0.927–0.992	0.851	0.614–0.948

a The maximum transverse dimension of the thyroid gland.

b The maximum longitudinal scan plane of the left thyroid lobe.

c The longitudinal scan plane of the left thyroid lobe.

d The maximum longitudinal scan plane of the right thyroid lobe.

e The oblique scan plane of the left thyroid lobe.

f The oblique scan plane of the right thyroid lobe.

g Length; craniocaudal dimension.

h Height; dorsoventral dimension.

i Width; mediolateral dimension.

j Cross-sectional area.

k Intraclass Correlation Coefficient.

l Confidence Interval.

m Portable ultrasound unit.

n Fully-equipped clinical ultrasound unit.

## Discussion

Ultrasound is considered as a safe, non-invasive and well-tolerated imaging method in non-sedated animals [19]. Diagnostic ultrasound enables serial examinations to monitor the progress of clinical condition and treatment response. The results of the present study demonstrated that ultrasound is an effective and reliable tool for measuring thyroid parameters. To the best of our knowledge, there has been no previous research investigating dolphin thyroid measurements using 2 different ultrasound machines, therefore the current study reflects the potential of detecting changes that exceed measurement error, for clinical and research applications.

There was a high level of agreement between the 2 ultrasound units in dolphin thyroid measurements, with the ICC values ranging from 0.859 to 0.976. Theoretically, the reproducibility (ICC) has a maximum value of 1. In most papers, a reproducibility of 0.7 and higher for labeling methods or units is considered to be sufficient [21], [22]. Thus, the results supported a high degree of agreement between the PUS and FCUS to quantify dolphin thyroid volume.

Results of the present study demonstrated that both the PUS and FCUS had a high intra-operator repeatability in thyroid measurements, with the ICC values of the PUS ranging from 0.854 to 0.984, and the ICC values of the FCUS ranging from 0.709 to 0.954. These results supported that the measurements yielded by the PUS are not only comparable to that of the FCUS, but that each unit can be used to perform thyroid volume measurements in a consistent manner.

Overall, the inter-equipment and intra-operator variability was minimal due to a number of reasons. The presence of a well-defined capsulated thyroid gland improved visualization on ultrasound scanning, enabling a higher precision while performing linear and cross-sectional area measurements. Since the dolphin thyroid gland was situated at the thoracic inlet, midway between the insertions of the pectoral flippers, this minimized measurement variation caused by the effect of physiological activity such as heart beats and breathing during the scan. In the present study, a standard scanning protocol for the four 2-D ultrasound thyroid volume measurement methods was implemented, allowing the operator to have a clear and a precise sense of the procedures, facilitating the consistency of measurements during the ultrasound scanning. A single operator performed the present study enabling familiarity and greater experience with the established protocol. All dolphins involved in the study were trained to cooperate for neck ultrasound examination in a dorsal recumbence position, with their neck straightened and remaining still at the poolside. This prevented the distortion of the thyroid gland and thus allowed higher consistency with measurements during the ultrasound scanning.

These findings are in accordance with the results of the previous *in vivo* and *in vitro* studies which have incorporated ICC as a statistical test to assess agreement. A high correlation in the inter-operator and intra-operator measurements of the mean splenic length (ICC value of 0.89 and 0.94) has been previously identified [23]; similarly, a high correlation was also demonstrated in the inter-operator and intra-operator measurements of the cross-sectional area of the tibial nerve at the tarsal tunnel (ICC values≥0.86) [24]. For inter-equipment variability, previous studies reported that measures obtained using both PUS and FCUS were not significantly different and were equally repeatable [25]–[27]. However, the direct comparisons must be treated with caution. Our present study focused on the agreement between the 2 compared ultrasound units, rather than the accuracy of the portable ultrasound unit itself. Comparison of dolphin thyroid volume measurement accuracy using the 2 captioned ultrasound units is not possible due to the lack of a standard of reference. In our previous study, 3-D ultrasound thyroid volume measured by the FCUS was compared with the 2-D ultrasound thyroid volume measurement with the identical ultrasound unit and settings [15]. 3-D ultrasound thyroid volume measurements cannot be used as the standard of reference in the present study, since 3-D ultrasound is a functional capability of the FCUS. The PUS measurements have a substantially different image quality, and thus would result in a bias in favour of the FCUS measurements. As such, instead of looking into the accuracy of both ultrasound units on their own, the present study investigated the agreement between these 2 ultrasound units (with the FCUS measurement accuracy validated in our previous study).

In the present study, the PUS yielded a higher intra-operator repeatability than the FCUS. Compared to the FCUS, the PUS has less precise calipers, limiting the measurements to 1 decimal place. In contrast, the FCUS gives the measurements to 2 decimal places, making it less prone to rounding error. This may give the PUS a higher intra-operator repeatability since the measurements had a higher degree of estimation with more measurements demonstrating absolute agreement.

The cross-sectional area measurements were found to have a higher inter-equipment reproducibility and intra-operator repeatability than that of the linear measurements. In a previous study, the cross-sectional area measurements of custom-made tissue phantoms had a higher inter- and intra-operator reliability than the linear measurements [28]. Additionally, the inter-operator variability for calculating thyroid volume was found to be statistically significant when using the formula with linear measurements, but was not statistically significant when using the formula with cross-sectional area measurements [14]. In the present study, for Methods A and B, the maximum cross-sectional area measurements from all 3 maximum longitudinal dimension scan planes yielded a higher reliability than the linear measurements (craniocaudal and dorsoventral dimensions). However, there may be difficulties in consistently estimating the linear measurements on the maximum longitudinal dimension scan plan between the 2 ultrasound scans. Since the thyroid gland was not a true oval shaped structure for the measurement on the longitudinal planes in Methods A and B and the transverse planes in Methods C and D, the determination of maximum long axis dimension was highly subjective, which possibly resulted in a larger variation on the linear measurements. In contrast, the determination of the maximum cross-sectional area relied on manual free-hand tracing of the thyroid borders, which was considered to be a relatively easier and more straight-forward procedure, resulting in a higher reproducibility and repeatability on the measurements. The same issues applied for Methods C and D, in which the maximum cross-sectional area measurements in the scan plane 90 degrees to the craniocaudal dimension also yielded a higher reliability than the linear measurements (mediolateral and dorsoventral dimensions). Moreover, it is possible that there are different measurements of the craniocaudal and dorsoventral dimensions on the same image plane; however, the cross-sectional area based on the same image plane would not change, resulting in a higher reliability than the linear measurements.

Even though this study has the undeniable merit of offering valuable insight into the agreement between the PUS and the FCUS in the application of dolphin thyroid measurements, there are some limitations. Due to the limited availability of multiple units, the number of unit representing in each category (PUS and FCUS) for comparison was restricted to one only. It may alter the results yielded using different units. Further studies in investigating the agreement with multiple units representing each category are suggested to minimize the intrinsic differences in the compared units. The transducers of the compared units were not in the identical frequency range. This is virtually unattainable since the FCUS in this study utilizes the latest transducer technology, which provides a broad range of frequencies rather than a single frequency emitted by the PUS compatible transducer. Image resolution may be degraded due to the frequency differences, and thus may affect the measurement accuracy. To minimize this difference in technology, the transducer frequency of the FCUS was set to the “middle to high” range between 5–2 MHz, which should be comparable to the 5 MHz used in the PUS transducer. With broad bandwidth transducers used in FCUS unit, the manipulation of transmit frequency bandwidth and received frequency bandwidth was allowed, which facilitated the operator to optimize image data to match the target requirement. ‘Middle to high’ frequency on the 5–2 MHz transducer of the FCUS unit was equivalent to 4.25 MHz centre frequency (3.5–5 MHz operational sensitivity). In addition, the issue of image quality comparison between the captioned ultrasound units had not been mentioned in the present study. According to a previous study, the image quality is undoubtedly a component of the diagnostic ability of a system, but is only one facet in determining an optimal system [29]. Although we believe that the measurement accuracy may possibly be affected by the different image quality yielded, the degree of influence should be insignificant in our case, due to the presence of a well-defined capsulated thyroid gland in the dolphin which allows for an accurate linear measurement on different thyroid dimensions. Despite the controversy in objectively defining the image quality [30], [31], there is no doubt that differential diagnosis was confirmed when a more advanced clinical ultrasound unit was used, which inevitably produced higher quality ultrasound images for clinical diagnosis. Studies have suggested that PUS provides a significant benefit that can drastically alter the disposition and treatment in patients at Accident and Emergency Departments, Intensive Care Units, small-scale hospitals and remote location settings [30], [32]–[34]. In view of the concerns raised from zoological and aquarium settings, a PUS could play an adequate role in improving a variety of veterinary procedures by providing a real-time, non-invasive clinical tool. Further studies in objectively evaluating the difference in image quality between the PUS and the FCUS in a zoological or aquarium setting are suggested to reinforce confidence of using PUS in veterinary medicine.

### Conclusions

There was no substantial inter-equipment variability between PUS and FCUS in thyroid size measurements. Both systems had high intra-operator repeatability in thyroid size measurements, substantiating further application of PUS for quantitative analyses of dolphin thyroid gland in research and clinical practice at an aquarium setting, when FCUS is not available.
